# Terbium-149 production: a focus on yield and quality improvement towards preclinical application

**DOI:** 10.1038/s41598-024-53610-2

**Published:** 2024-02-08

**Authors:** C. Favaretto, P. V. Grundler, Z. Talip, U. Köster, K. Johnston, S. D. Busslinger, P. Sprung, C. C. Hillhouse, R. Eichler, R. Schibli, C. Müller, N. P. van der Meulen

**Affiliations:** 1 Nuclear Medicine Department, University Hospital Basel, Basel, Switzerland; 2 Center for Radiopharmaceutical Sciences ETH-PSI, Paul Scherrer Institute, 5232 Villigen-PSI, Switzerland; 3 Institute Laue-Langevin, Grenoble, France; 4 Physics Department, ISOLDE/CERN, Geneva, Switzerland; 5 Department Hot Laboratory, Paul Scherrer Institute, Villigen-PSI, Switzerland; 6 Laboratory of Radiochemistry, Paul Scherrer Institute, Villigen-PSI, Switzerland; 7 Department of Chemistry, Biochemistry and Pharmaceutical Sciences, University of Bern, Bern, Switzerland; 8 Department of Chemistry and Applied Biosciences, ETH Zurich, Zurich, Switzerland

**Keywords:** Analytical chemistry, Nuclear chemistry

## Abstract

Terbium-149 (T_1/2_ = 4.1 h, E_α_ = 3.98 MeV (16.7%), 28 µm range in tissue) is a radionuclide with potential for targeted alpha therapy. Due to the negligible emission of α-emitting daughter nuclides, toxicity to healthy tissue may be reduced in comparison with other α-particle emitters. In this study, terbium-149 was produced via 1.4 GeV proton irradiation of a tantalum target at the CERN-ISOLDE facility. The spallation products were mass separated and implanted on zinc-coated foils and, later, radiochemically processed. Terbium-149 was separated from the co-produced isobaric radioisotopes and the zinc coating from the implantation foil, using cation-exchange and extraction chromatographic techniques, respectively. At the end of separation, up to 260 MBq terbium-149 were obtained with > 99% radionuclidic purity. Radiolabeling experiments were performed with DOTATATE, achieving 50 MBq/nmol apparent molar activity with radiochemical purity > 99%. The chemical purity was determined by inductively coupled plasma–mass spectrometry measurements, which showed lead, copper, iron and zinc only at ppb level. The radiolabeling of the somatostatin analogue DOTATATE with [^149^Tb]TbCl_3_ and the subsequent in vivo PET/CT scans conducted in xenografted mice, showing good tumor uptake, further demonstrated product quality and its ability to be used in a preclinical setting.

## Introduction

The concept of targeted radionuclide therapy towards the treatment of cancer has gained more attention recently, however, predominantly for late-stage disease. While this type of therapy has focused mainly on the use of β^−^-emitters, Targeted Alpha Therapy (TAT) has garnered recent interest due to the promising results obtained with ^225^Ac-based radionuclide therapy of cancer patients^1–4^. Alpha-particles emitted from radionuclides have a high linear energy transfer (LET: ~ 80 keV/μm) and a short tissue-penetration range (40–100 µm)^5^. These characteristics make α-emitting radionuclides attractive for the treatment of microscopic metastases that are responsible for relapse of the disease. However, each α-decay is accompanied by an energetic recoil nucleus and, as a result, the recoil daughter will break chemical bonds with the biomolecule, possibly even destroying the chelator, resulting in the release of the daughter radionuclide^6^. The clinical use of actinium-225 may, thus, be questioned due to potential long-term undesired side effects to radiosensitive organs and tissue, in which the (α- and β^−^-particle-emitting) daughter nuclides of actinium-225 may accumulate. In addition, due to the different chemical nature of the decay daughters starting from francium as an alkali metal, over astatine as metalloid halogen to the heavy metals bismuth and polonium, the accumulation is difficult to predict and to prevent.

**Figure 1:**
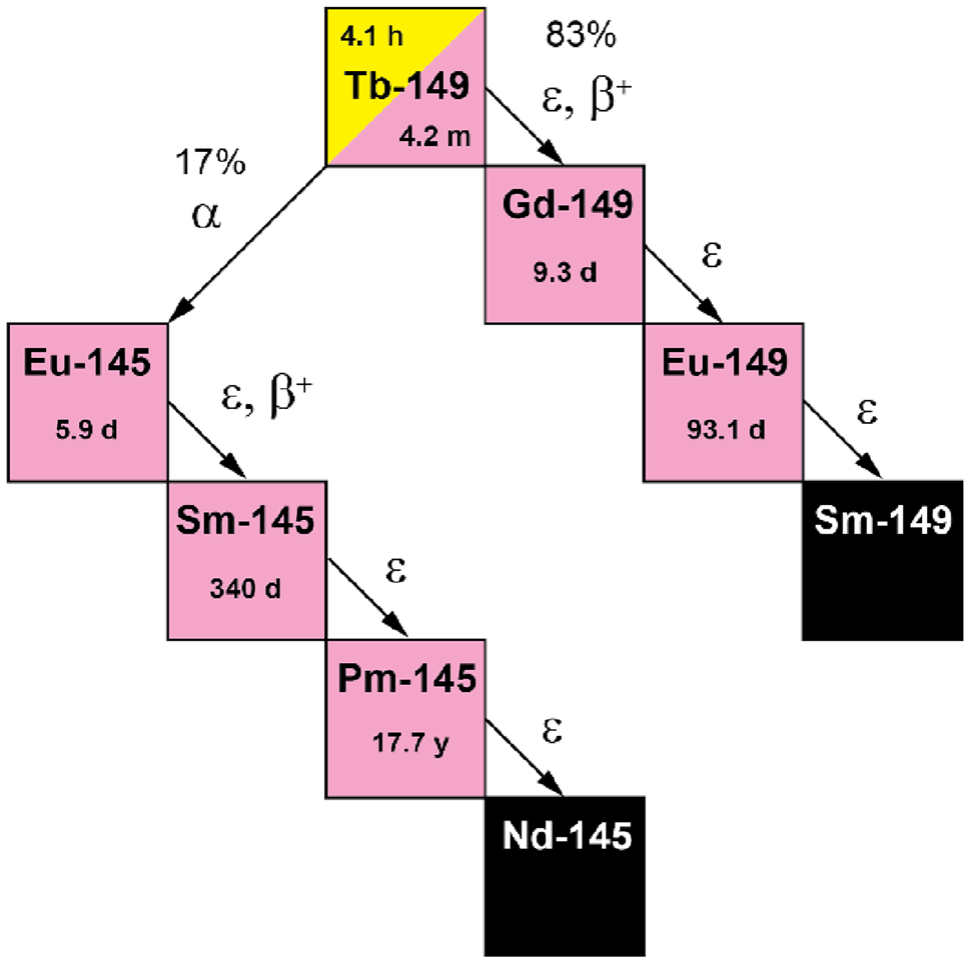
Simplified decay scheme of terbium-149.

The suggested use of terbium-149 was proposed for TAT in the late 1990’s, at a similar time to when ^225^Ac/^213^Bi generators were introduced^7^. Terbium-149 is a partial α-emitter (T_1/2_ = 4.1 h, E_α_ = 3.98 MeV (16.7%), 28 µm range in tissue), while the remaining 83.3% of the decay occurs through electron capture (ε) and positron emission (E_β_^+^_average_ = 720 keV, I = 7.1%) respectively^8,9^. Although actinium-225 has been favored due to its comparatively ease of accessibility, terbium-149 may still be regarded as an another interesting α-emitter thanks to its daughter nuclides that emit a negligible number of α-particles (< 0.001%), thus, possibly being advantageous with regard to the safety profile in patients (Fig. 1)^8,10^. Finally, terbium-149's half-life lies between those of bismuth-213 (T_1/2_ = 45 min)^11^ and actinium-225 (T_1/2_ = 9.9 d)^12^ and is about half that of astatine-211 (T_1/2_ = 7.21 h)^13^.

To date, only a small number of preclinical proof-of-concept studies involving terbium-149 have been published^9,14–16^. Beyer et al. performed preclinical experiments, in which ^149^Tb-labeled rituximab was applied in a leukemia mouse model, producing promising results^14^, while, at Paul Scherrer Institute (PSI), terbium-149 was successfully investigated in additional preclinical studies using a DOTA-folate ligand^16^ and, more recently, PSMA-617^15^. Terbium-149 is also attractive because of its emission of β^+^-particles, which enables imaging by means of positron emission tomography (PET). This attribute was demonstrated using [^149^Tb]Tb-DOTANOC and [^149^Tb]Tb- PSMA-617 in mouse models of somatostatin receptor-expressing tumors and PSMA-positive tumors, respectively^9,15^. However, the application of imaging activity might have associated therapeutic effects, thereby limiting its applicability to treatment monitoring rather than pre-treatment dosimetry and diagnosis. Nevertheless, terbium isotopes have been acknowledged for their potential in radiotheragnostics, with four radioisotopes that are of medical relevance: terbium-149 for α-therapy, terbium-152 for PET, terbium-155 for Single-Photon Emission Computed Tomography (SPECT), and terbium-161 for β^−^-therapy^17^. Therefore, the utilization of terbium-149 would be optimal for therapeutic purposes and monitoring during therapy, while the diagnosis or pre-treatment dosimetry could be conducted with the same targeting vector, radiolabeled with an imaging terbium radioisotope. This would accurately reflect the concept of “matched pair” radioisotopes in nuclear oncology^18^.

Despite the demonstration of terbium-149’s efficacy, preclinical research with the radionuclide is still in its infancy due to its limited availability. Isotopically pure terbium-149 samples were first produced by 65 MeV proton irradiation of natural gadolinium oxide, followed by radiochemical separation of the terbium fraction and subsequent offline mass separation^19^. Later, cross-sections for proton-induced reactions were determined on 30% enriched gadolinium-152 targets^20^. However, the overlapping excitation curves of neighboring terbium radioisotopes were found, limiting the achievable radionuclidic purity (RNP) (< 65%). In addition, the manufacturing of highly enriched gadolinium-152 targets is technically feasible, but remain fundamentally challenging due to gadolinium-152's low natural abundance (0.2%)^21^. ^151^Eu(^3^He,5n)^149^Tb or ^151^Eu(^4^He,6n)^149^Tb reactions on enriched europium-151 targets (47.8% natural abundance) were proposed, but again co-production of neighboring terbium radioisotopes led to low RNP^22,23^. Various heavy-ion induced reactions leading to terbium-149 have been discussed; among them, ^12^C-irradiations of neodymium targets were tested experimentally; however, the low RNP and the lack of accelerators providing intense carbon ion beams of suitable energy (≈10 MeV/nucleon) limit the method applicability^24,25^. Finally, spallation of heavy target materials (e.g. tantalum, tungsten or gold) induced by high-energy protons (at energies between about 600 MeV and 3 GeV) produced terbium-149, as well as other terbium radioisotopes^24,26,27^. In summary, all known production routes for terbium-149 lead to co-production of other terbium radioisotopes, which leads, in turn, to a RNP that is generally insufficient for medical applications. Thus, an additional mass-separation step has to be introduced in the separation scheme^28^. In principle, this could be applied to terbium-149 produced by any of the mentioned reactions in an offline process, i.e. first irradiation, then separation. However, in practice, it is most convenient to perform irradiation and separation simultaneously with the so-called Isotope Separation On-Line (ISOL) process, using reusable high-melting targets^29^. For over five decades, CERN-ISOLDE has employed high-energy proton beams on tantalum-foil targets to produce and selectively collect isotopes with mass 149^30^^,^^31^. It is worth noting that the majority of collected terbium-149 activity results from the decay of dysprosium-149 ions (T_1/2_ = 4 min)^8^ that are released more easily than terbium. In addition, the implementation of resonant laser ionization (RILIS) in recent years enhanced the ionization efficiency of dysprosium over that of other isobars in the beam^32^. However, after the ISOL production, a chemical separation is still needed to obtain a radionuclidically and chemically pure product.

In this work, the production of terbium-149, via the abovementioned spallation and online mass separation, was combined with radiochemical separation to provide a good-quality product for use in preclinical studies. The purity was proven by γ-ray spectrometry, Inductively Coupled Plasma–Mass Spectrometry (ICP-MS) measurements and radiolabeling experiments. The product quality and its suitability for preclinical studies was further demonstrated with in vivo PET/CT scans, depicting uptake of [^149^Tb]Tb-DOTATATE on somatostatin-positive tumor-bearing mice.

## Materials and methods

### Irradiation conditions and online mass separation

Terbium-149 was produced by proton-induced spallation of tantalum targets, using the online isotope separator facility ISOLDE at CERN (Geneva, Switzerland), as previously reported^15,17^. A tantalum target (94 g/cm^2^) was irradiated with 1.4 GeV protons from the CERN PS-Booster accelerator. The spallation products were released from the target material, kept at ~ 2100 ºC, and resonantly laser ionized with a two-step and three-step RILIS scheme used in parallel^32–35^. The resulting singly-charged ion beam was accelerated to 30 keV and mass-separated in a magnetic sector field. Mass 149 isobars and molecular pseudo-isobars were implanted into zinc-coated (prepared by physical vapor deposition, 0.625 mg/cm^2^) gold or platinum foils (8 mm × 40 mm, 0.1 mm and 0.025 mm thick, respectively) over a period of 6 to 9 h. This process was followed by 1.5 h of “cooling” to allow short-lived radionuclides to decay. The irradiations and collections were performed overnight, over four separate one-week campaigns. During the initial two campaigns, the same tantalum target was used, whereas a new target was implemented from the third campaign. After the irradiation and collections, the zinc-coated foils were shipped the following morning to PSI, where the radiochemical separation was started upon arrival to deliver purified terbium-149 for further experiments by late afternoon.

### Radiochemical separation

#### Development of the separation process

The method for terbium-149 separation from the isobaric radiolanthanides, as well as the zinc from the implantation foil, was developed by means of bench experiments using stable isotopes of the elements of interest. The lanthanides investigated in this development work were selected based on previous characterizations of implanted gold foils^36^ and on the following criteria. Terbium, gadolinium, europium (as A = 149 isobaric contaminants, Supplementary Table S1)^8^, and cerium (as A = 133 pseudo-isobar contaminants in the ^133^Ce^16^O^+^ form, Supplementary Table S2)^37^ were included in the separation development process due to their decay time (T_1/2_ > 1.5 h) and highly similar chemical behavior, thus, necessitating a separation from the desired product. The pseudo-isobar praseodymium-133 oxide (^133^Pr^16^O^+^) was not considered, due to the short half-life of praseodymium-133, which would have resulted in its decay by the time of delivery to PSI (Supplementary Table S2)^37^. Additionally, lanthanum was excluded from the research due to its expected delayed elution in comparison to cerium during the separation process. Finally, dysprosium was also not considered in this study, as dysprosium-149 is the parent radionuclide of terbium-149 with a short half-life of only 4.2 min (Supplementary Table S1)^8^.

To simulate the sample obtained by dissolving the zinc coating on the implantation foil at the end of collection, a stock solution was prepared as follows. Aliquots of 50 µL from commercial ICP standard solutions of terbium, gadolinium, europium and cerium, each with 1000 ppm of the respective lanthanide in HNO_3_ (2–3%), were mixed together with 20 mg of zinc dissolved in 12 mL 0.5 M HNO_3_. The large excess of zinc enabled the development of a separation method that can effectively operate under conditions of high zinc content, thereby, ensuring the successful separation even in more challenging circumstances than those expected for the radioactive zinc-coated gold foil. The solution was neutralized with 5.0 mL 1.0 M NH_3_ (Suprapur, Merck GmbH, Germany) and loaded onto a chromatographic column (6 mm × 50 mm), containing 1.4 mL Sykam macroporous cation exchange resin (Sykam Chromatographie Vertriebs GmbH, Germany; particle size 12–22 μm, NH_4_^+^ form, column volume: 1.4 mL), using a peristaltic pump. The dimension of the column was selected considering the low mass of lanthanides to be separated, with the goal of achieving the shortest possible separation time to minimize activity loss due to the decay of terbium-149. Afterwards, the column was rinsed with 22 mL high purity water (resistivity > 18 MΩ). The column was further rinsed with 20 mL 1.0 M NH_4_NO_3_ (Suprapur, Merck GmbH, Germany) at a flow rate of 0.6 mL/min and the eluted volume collected in 5 mL fractions. The lanthanide separation was performed on the Sykam column by gradient elution with the use of α-hydroxy-isobutyric acid (α-HIBA, Sigma-Aldrich GmbH, Germany) as eluent (0.6 mL/min flow rate). Five mL 0.07 M α-HIBA (pH 4.5) were passed through the Sykam column, followed by further 5 mL 0.09 M α-HIBA (pH 4.5) and 50 mL 0.11 M α-HIBA (pH 4.5). The concentration of α-HIBA was determined in line with elution profiles obtained for other terbium separations developed by our group^38,39^. Significant optimization steps involved introducing a gradient elution to improve lanthanide separation and implementing the initial NH_4_NO_3_ column rinse to enhance zinc separation (Supplementary Fig. S1). Finally, the column was cleaned using 15 mL 1.0 M α-HIBA followed by 15 mL 4.0 M HNO_3_ as the final cleaning step. Samples (5 mL fractions) of α-HIBA were collected during the entire process. The concentration of the lanthanides in the 5-mL fractions was measured by means of Inductively Coupled Plasma–Optical Emission Spectrometry (ICP-OES, Agilent 5110 ICP-OES, USA). With the data gathered from these experiments, the elution profiles of zinc and the investigated lanthanides from Sykam resin were established.

#### Separation of terbium-149

**Figure 2:**
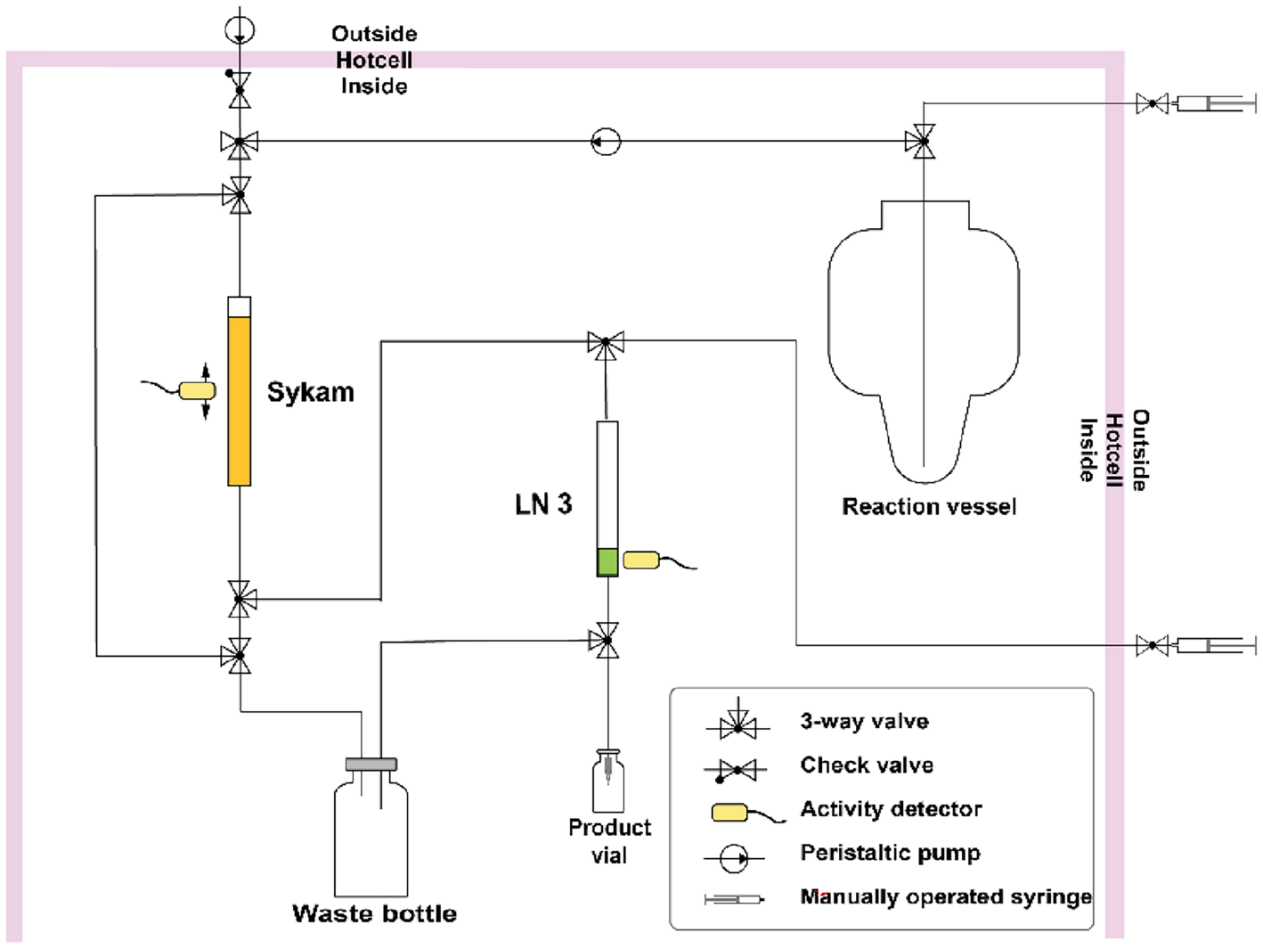
Schematic diagram of the terbium-149 separation panel. Orange and green colors in the diagram represent the columns that contain Sykam and LN3 resins, respectively.

The radiochemical separation method and the resultant separation system (Fig. 2), designed to process the zinc-coated foils used for the implantation of terbium-149, were established according to the results obtained with stable isotopes as described in the previous section. The system was placed into a designated shielded cell and the radiochemical separations of terbium-149 were performed with the use of manipulators, such that high activities could be processed with minimal radiation dose exposure to the operator.

During the campaigns, each zinc-coated implanted foil, received at PSI from CERN-ISOLDE, was transferred into the separation system and the zinc layer containing the radiolanthanides was dissolved in the reaction vessel with 12 mL 0.5 M HNO_3_. Once the zinc coating had completely dissolved, 5.0 mL 1.0 M NH_3_ were added to achieve pH ~ 1.5. A ~ 50 µL aliquot of the solution was withdrawn from the reaction vessel, before the separation, to be measured by γ-ray spectrometry. The remaining solution was loaded at a flow rate of 0.6 mL/min onto a chromatographic column filled with Sykam macroporous cation exchange resin (same column size as used in the method development). The separation with the Sykam column was performed with α-HIBA gradient elution, according to the method described with stable isotopes in the previous section. During the entire process, the movements of the radiolanthanides along the column was monitored by means of Si PIN-diodes, which acted as detectors. When activity was detected at the bottom of the cation exchange column, the eluate was directed onto a second column, containing 0.08 mL bis(1,4,4-trimethyl-1-pentyl)phosphinic acid extraction resin (LN3, Triskem International, France; particle size 50–100 μm). The terbium-149 product was finally eluted from the second column in 1 mL 0.05 M HCl, in analogy to the terbium-161 and terbium-155 radiochemical separation methods previously developed by our group^38,39^. The pH of the final product was determined using pH indicator strips.

### Quality controls

#### Production yield and RNP

The 50 µL aliquot withdrawn from the reaction vessel at the beginning of the separation and an aliquot of the [^149^Tb]TbCl_3_ solution obtained at the end of separation (EOS) were measured by γ-ray spectrometry using a high-purity germanium (HPGe) detector (Canberra, France) featuring the Inter-Winner software package (version 7.1, Itech Instruments, France). The efficiency calibration was performed using an Eppendorf vial filled with a europium-152 solution (78.96 kBq ± 0.80%, reference date 20.02.2017) with a source-detector distance of 1 m. In the same way, the specified aliquots were measured in similar Eppendorf vials as the europium-152. An uncertainty of ≤ 5% on the activity was estimated for each of the radionuclides. Thus, the contaminants could be determined before the separation as well as the activity and RNP at EOS was ascertained. The implantation foils were also measured using the same method after the dissolution of the zinc layer.

#### Chemical purity

The chemical purity of five aliquots of [^149^Tb]TbCl_3_ collected at EOS from four individual productions was assessed using an Element II® Sector Field ICP-MS (Thermo Fisher Scientific, USA). The samples were analyzed for contents of critical metallic contaminants according to European Pharmacopeia (e.g. lead, iron, copper, zinc^40^), and of several lanthanides, employing the low (gadolinium, terbium, dysprosium, lead) and medium mass resolution (iron, copper, zinc) setting, a cyclonic perfluoroalkoxy alkanes (PFA-Teflon®) spray chamber, a “PFA Microflow Nebulizer” (Elemental Scientific) and a peristaltic pump achieving a constant analyte consumption rate of ~ 50 μL/min. The plasma was operated in hot-plasma mode (RF power 1350W) and a discrete dynode secondary electron multiplier was used as a detector. The machine was routinely tuned before every sequence measurement for optimal sensitivity (ppb boron, rhodium, and uranium) at minimum oxide formation rates (UO/U < 3%). The determination of the element concentrations in the samples was performed by external standard calibration with a certified multi element standard (TraceCert Mix 1 and Mix 3). Samples and standards were prepared in 0.028 M HNO_3_ (67–70% SCP Science PlasmaPURE Plus). All dilutions were made gravimetrically (Mettler Toledo XP56 Micro-Analytical-Balance). A mixed cobalt-holmium-rhenium-standard (gravimetrically mixed to ca. 1:1:1, stock: 10 mg/L in 2% HNO_3_, Elemental Scientific) was added gravimetrically to all samples and standards as an internal standard at a concentration of approximately 50 ppb. The samples were measured once, due to the limited amount of material available, with the standard solutions being measured at the beginning and end of each sequence. Instrumental background signals were defined by the preceding and intermittent analyses of 0.028 M HNO_3_, subtracting them from each individual measure.

#### Radiolabeling yield

Terbium-149 in 0.05 M HCl solution was mixed with 0.5 M sodium acetate to achieve a final pH of ~ 4.5^41^. DOTATATE (stock solution 1 mM) was added to obtain [^149^Tb]Tb-DOTATATE at an Apparent Molar Activity (AMA) of up to 50 MBq/nmol. The reaction mixture was incubated at 95 °C for 20 min. The radiolabeling yield was determined by means of high-performance liquid chromatography (HPLC, Merck Hitachi LaChrom HPLC system equipped with a D-7000 interface, a radioactivity detector (LB 508, Berthold Technologies GmbH) and a L-6200A pump using a reversed-phase C18 column (5 µm, 4.6 × 150 mm, Xterra™, Waters, USA)). A linear gradient of 0.1% TFA in high-purity water (95–20%) and acetonitrile (5–80%) over 15 min was used as mobile phase at a flow rate of 1 mL/min. The sample for the analysis consisted of ~ 0.5 MBq of the radiolabeling solution in high-purity water containing pentasodium diethylenetriamine pentaacetic acid (Na_5_-DTPA, 50 μM). For the estimation of the radiochemical purity (RCP) of [^149^Tb]Tb-DOTATATE from the HPLC chromatogram obtained, the fraction of the integrated product peak was calculated relative to the sum of all integrated radioactive peaks (the radiolabeled product, potentially released radionuclide subsequently bound to DTPA, as well as degradation products of unknown structure), which were set as 100%.

### Preclinical PET/CT scan

The animal experiments were carried out according to the ARRIVE guidelines and the Swiss Regulations for Animal Welfare, ethically approved by the Cantonal Committee of Animal Experimentation and permitted by the responsible cantonal authorities (license N° 79692).

Five-week-old female athymic CD1 nude mice (Crl:CD1-*Foxn*^*nu*^) were subcutaneously inoculated with AR42J tumor cells, a rat pancreatic tumor cell line, on the right shoulder as previously reported^42^. PET/CT scans were acquired 10–14 days after tumor cell inoculation when the xenografts reached an average size of ⁓500 mm^3^. [^149^Tb]Tb-DOTATATE (5 MBq, 0.5 nmol, prepared as described above and diluted in PBS with 0.05% bovine serum albumin) was injected into a lateral tail vein of the mouse (n = 2). The scans were performed using a small-animal bench-top PET/CT scanner (G8, PerkinElmer, Massachusetts, U.S.A^43^), as previously reported^15^, with a set energy window ranging from 150 to 650 keV. Static whole-body PET scans of 10 min duration were performed at 2 h after injection of the radiopeptide, followed by a CT scan of ⁓1.5 min (Supplementary information).

## Results

### Irradiation conditions and mass separation

**Figure 3:**
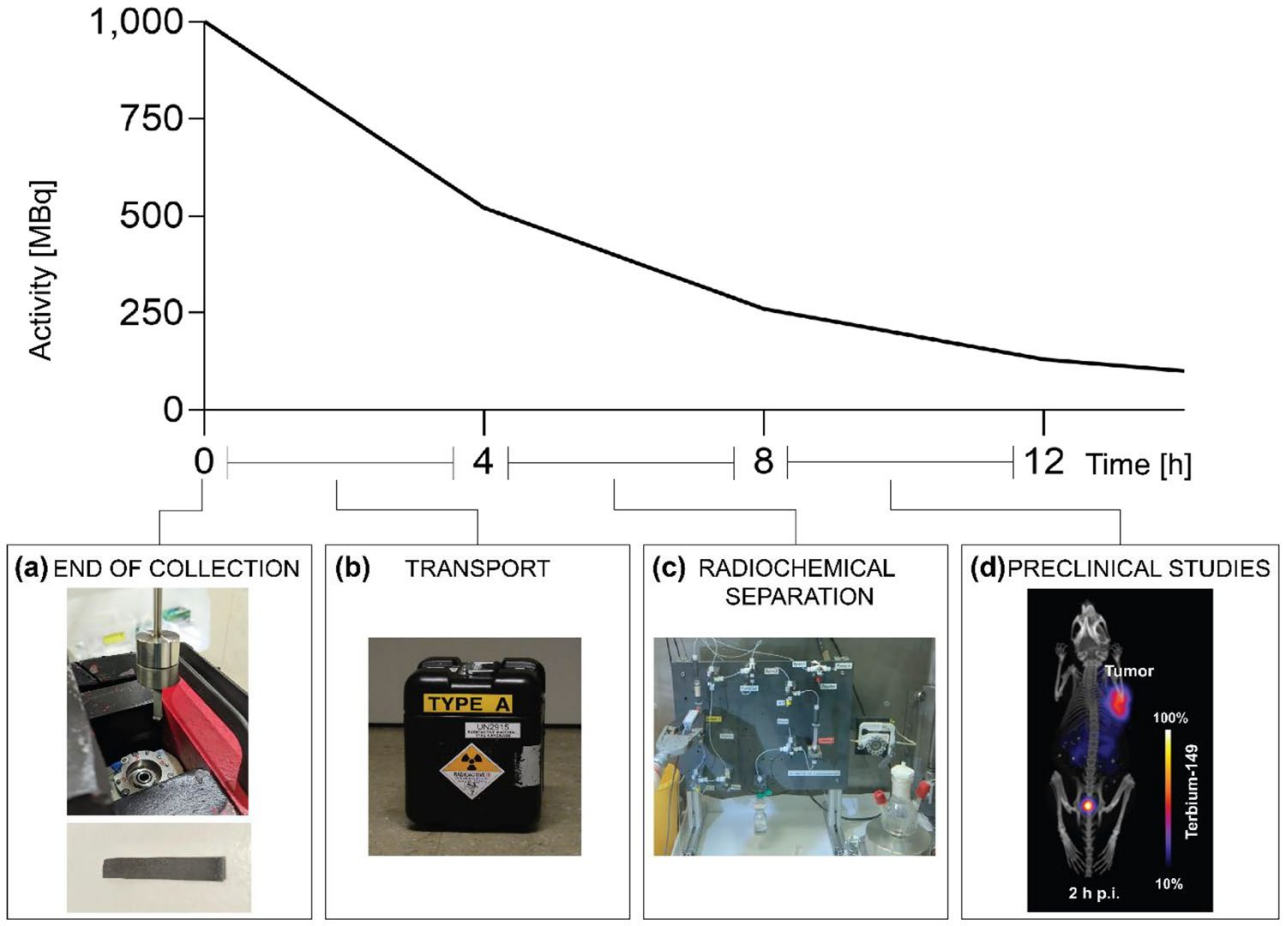
Timeline depicting the decay of terbium-149, along with the processes performed over the period: collection (implantation foil during installation in the collection chamber and isolated view of the implantation foil (**a**), transport (shielded transport box, (**b**), radiochemical separation (separation setup inside hot cell (**c**), and preclinical studies (mouse PET/CT scan with [^149^Tb]Tb-DOTATATE (**d**).

**Figure 4:**
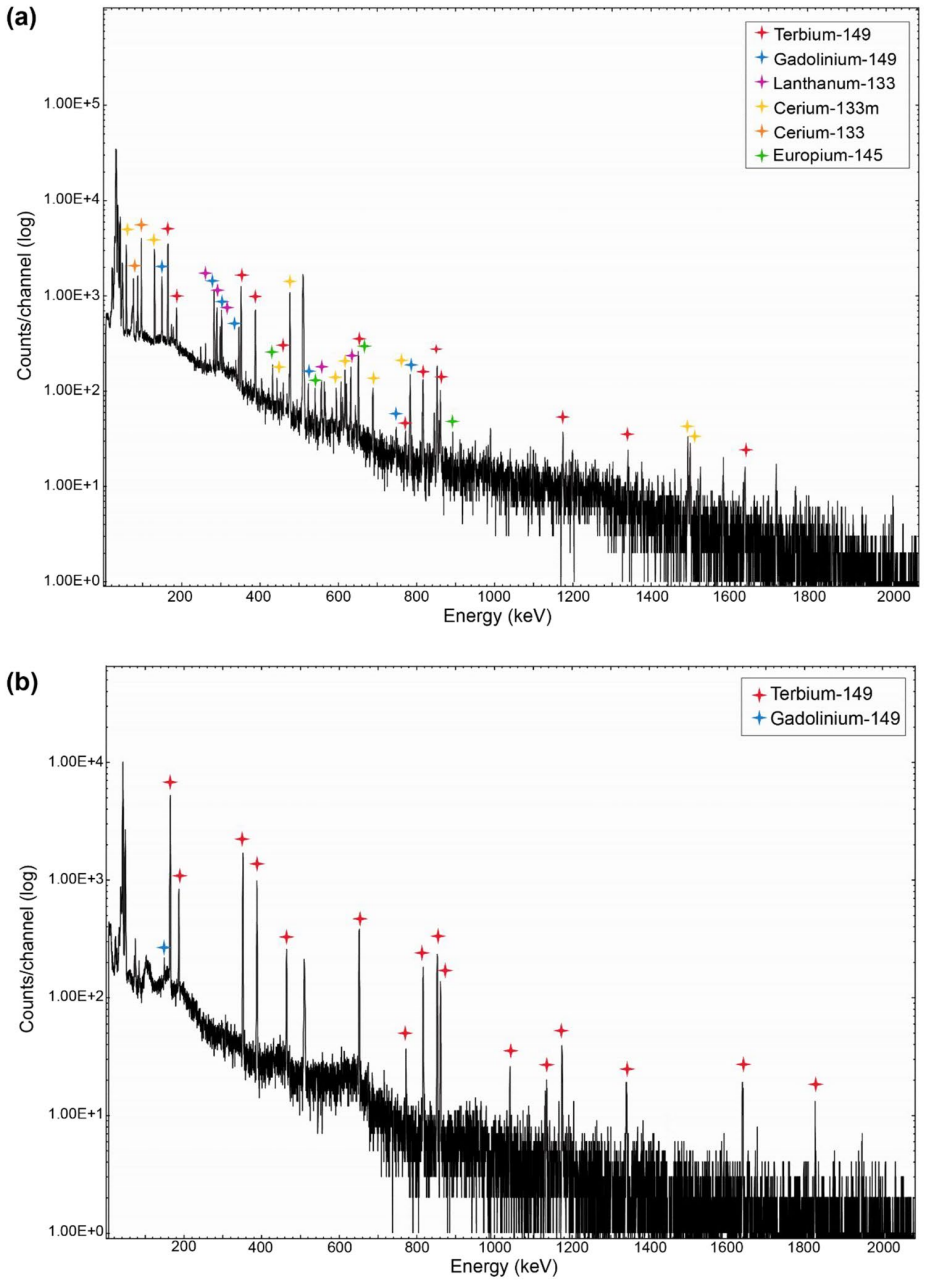
(**a**) Representative γ-spectrum of an aliquot taken from the initial terbium-149 solution before radiochemical separation (55-min measurement time; 1 m distance from the detector). Red star = terbium-149 peaks, blue star = gadolinium-149 peaks, violet star = lanthanum-133 peaks, yellow star = cerium-133 m peaks, orange star = cerium-133 peaks, green star = europium-145 peaks. (**b**) Representative γ-spectrum of terbium-149 obtained after the radiochemical separation process (60-min measurement time starting 11 min after EOS; 1 m distance from the detector; ~ 0.5 MBq). Red star = terbium-149 peaks (MDA 34 kBq), blue star = gadolinium-149 peak (MDA 2.4 kBq).

Four campaigns for terbium-149 production, conducted at CERN-ISOLDE, resulted in the collection of a total of 16 samples. According to the calculated implanted activities, up to ~ 1000 MBq of terbium-149 per day was sent to PSI. Due to the time taken to transport the sample from CERN to PSI, it was estimated that up to an activity of ~ 500 MBq arrived on site for processing (Fig. 3). For each sample, an aliquot of the dissolved zinc layer was measured, in which terbium-149, along with other A = 149 isobars and A = 133 oxide sidebands (or pseudo-isobars), were detected (Fig. 4a). In addition, europium-145 was measured, as it is one of the decay products of terbium-149 (Fig. 1).

### Radiochemical separation

#### Development of the separation process

**Figure 5:**
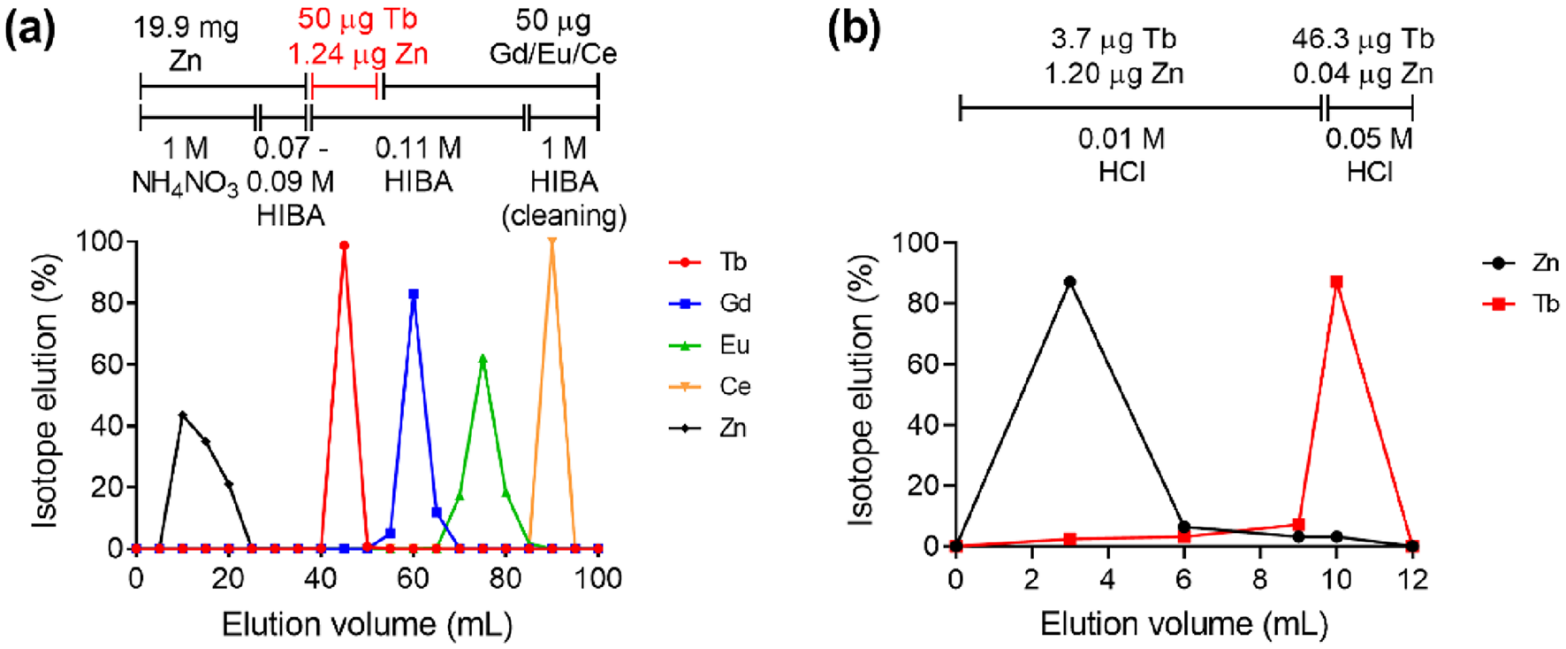
(**a**) Representative elution profile of zinc, terbium, gadolinium, europium and cerium from Sykam resin (1.4 mL column) using 20 mL 1.0 M NH_4_NO_3_ followed by gradient elution 0.07–1.0 M α-HIBA. (**b**) Elution profile of zinc traces and terbium from LN3 resin (0.8 mL column) using 10 mL 0.01 M HCl followed by 1 mL 0.05 M HCl.

With the data gathered during the bench experiments with stable isotopes, the elution profiles of zinc and the lanthanides expected to be collected as isobaric impurities were established for the 1.4 mL cation exchange resin (Sykam resin) column proposed for the radiochemical separation process (Fig. 5a). In particular, after loading, zinc was almost completely removed from the column with the 20-mL-NH_4_NO_3_ rinse step. The following gradient elution with α-HIBA on the cation exchange resin allowed the lanthanide separation to take place. Terbium was the first lanthanide eluted, followed by gadolinium and europium, while cerium was eluted only with the more highly-concentrated α-HIBA used for rinsing the column as the last step. The last traces of zinc were removed on the LN3 column and highly pure terbium was finally eluted in 1 mL 0.05 M HCl (Fig. 5b).

#### Separation of terbium-149

During the first campaign four samples were processed, while five samples were radiochemically separated during the second campaign. Three and four collections were processed in the third and fourth campaign, respectively. A maximum of 4 h was needed to complete the radiochemical purification process using the system described above (Fig. 3c). The separation procedure yielded solutions of terbium-149 in 0.5 to 1 mL of 0.05 M HCl (pH 1–2), which were typically eluted into two or three fractions to assess the potential variability of the quality during the final elution step.

### Quality controls

#### Production yield and RNP

The γ-ray spectra obtained from aliquots of [^149^Tb]TbCl_3_ solutions at EOS showed only γ-lines characteristic for terbium-149 and its decay product gadolinium-149 at the low activity level expected from its in-growth during the period of time between EOS and measurement, indicating the success of the separation process from the co-produced isobars (Fig. 4b). The RNP at EOS was determined to be > 99.8%. In addition, the final terbium-149 obtained was quantified and resulted in activities up to 260 MBq, with the activity concentrations ranging from 20 to 280 kBq/µL (Table 1). In addition, negligible amounts of residual activity were detected in the implantation foil at EOS (2–50 kBq; < 0.2% of total activity EOS) (Supplementary Fig. S2).

**Table 1:** Terbium-149 activities obtained at EOS.

Campaign	Production	^149^Tb EOS (MBq)	> 95% radiolabeled AMA to DOTATATE (MBq/nmol)
I	1	52	10
	2	109	10
	3	100	20
	4	87	20
II	1	43	10
	2	57	10
	3	56	10
	4	25	10
	5	38	10
III (new Ta target installed)	1	260	50
	2	188	20
	3	254	50
IV	1	195	20
	2	207	20
	3	255	20
	4	217	20

#### Chemical purity

**Figure 6:**
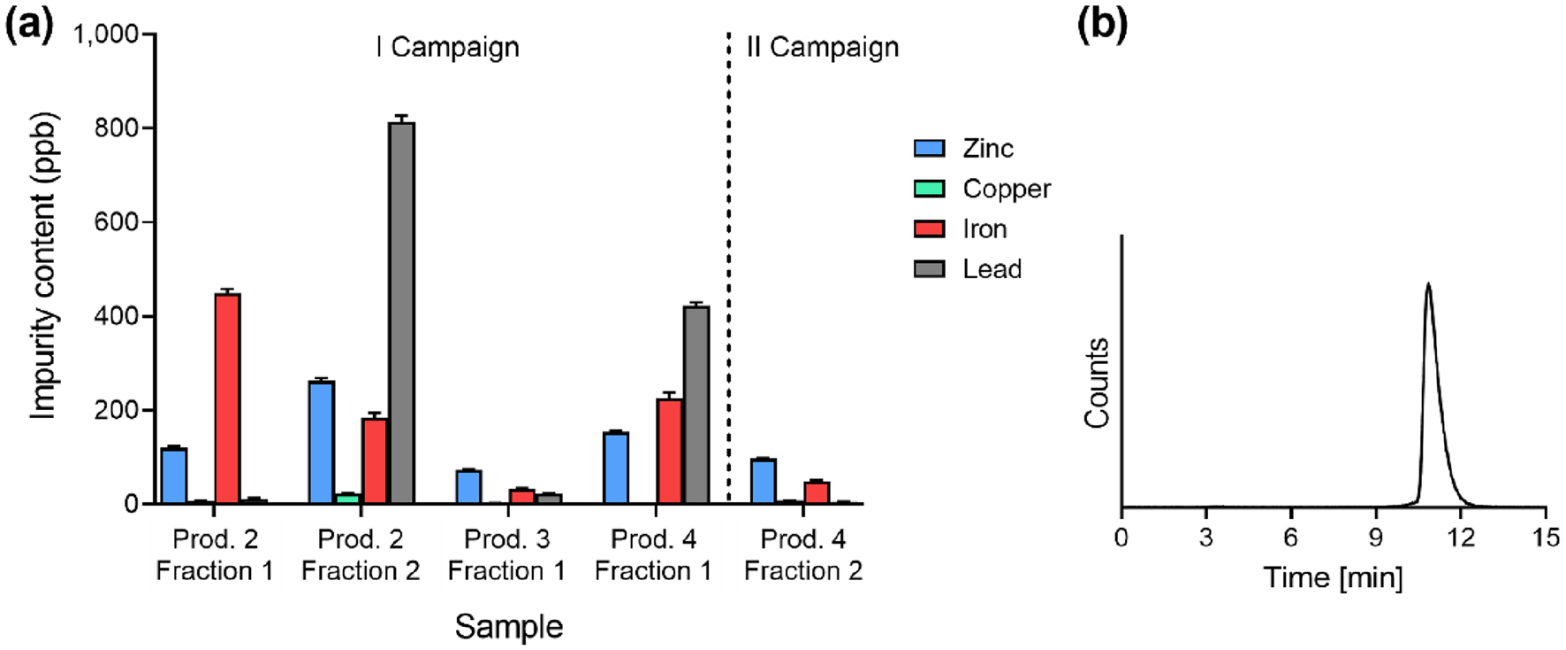
Quality controls of chemical purity of terbium-149. (**a**) Zinc, copper, iron and lead contents in 5 samples of [^149^Tb]TbCl_3_ at EOS. (**b**) Radiodetector signal of a representative HPLC chromatogram of 50 MBq/nmol [^149^Tb]Tb-DOTATATE (3.2 min retention time indicates “free” or unlabeled terbium-149, while 10.9 min indicates [^149^Tb]Tb-DOTATATE).

The contents of gadolinium, terbium and dysprosium were below detection limits (LOD < 4 ppb) in all the [^149^Tb]TbCl_3_ samples at EOS, while the critical metallic contaminants according to European Pharmacopeia (i.e. lead, iron, copper, zinc^40^) were measured in ppb levels (Fig. 6a, Supplemental Table S3).

#### Radiolabeling yield

As a further quality control, and to indirectly evaluate the chemical purity of the product, the radiolabeling yield of the [^149^Tb]TbCl_3_ solution was assessed to verify the capability of the radionuclide to be used for radiolabeling of biomolecules, as required for (pre)clinical use.

Radiolabeling of DOTATATE with terbium-149 was reproducible at 10 MBq/nmol AMA for all the fractions eluted at EOS, with > 99% RCP (Table 1). When the EOS activity was > 80 MBq, higher AMA, up to 50 MBq/nmol, was achievable with > 99% RCP (Fig. 6b). Higher molar activities could not be tested, due to the insufficient activity or activity concentration obtained.

### Preclinical PET/CT scan

PET/CT scans with AR42J tumor-bearing mice successfully visualized the accumulation of [^149^Tb]Tb-DOTATATE in the tumor tissue, while almost all activity was already cleared from the blood and kidneys at 2 h after injection with some residual activity seen in the urinary bladder (Fig. 3d). Therapy results obtained in this mouse model using [^149^Tb]Tb-DOTATATE will be presented elsewhere, together with in vitro studies performed to investigate the reduction in viability of AR42J tumor cells after exposure to ^149^Tb -based somatostatin analogues.

## Discussion

The present work demonstrated the efficacy of the spallation reaction Ta(p,spall)^149^Dy followed by online mass separation and the developed radiochemical separation method, to produce sufficient quantities of terbium-149 for preclinical studies. Compared with previous studies producing from a few MBq^17^ up to 25 MBq^15,16^ at EOS, the campaigns’ production yield was considerably improved, reaching up to tenfold enhancement. This improvement is attributed to the use of a new type of laser ion source with a modified construction, assuring a more efficient release and ionization of the short-lived dysprosium-149. However, despite the overall enhanced yields, due to logistics limitations (~ 4 h transport from CERN to PSI) and additional ~ 4 h for radiochemical separation, the final terbium-149 activity at EOS remains at about 25% of that collected. Hence, with more efficient logistics, these results could be further enhanced. In addition, as a result of the progressive aging of the tantalum target employed during the initial two campaigns, it was observed that these campaigns yielded less activity (particularly the second one) when compared to subsequent campaigns that were conducted following to the installation of a new target. Indeed, the tantalum target undergoes pulsed beam heating caused by the impact of protons, leading to accelerated sintering of the thin target. This phenomenon increases the delay losses of short-lived radionuclides^44^. In this study, zinc-coated gold or platinum foils were used to implant terbium-149 after production and mass separation. The use of zinc-coated foils, as compared to pure zinc foils previously tested^36^, enables the chemical separation from much smaller quantities of zinc (layers of a few hundred nanometers, 2 mg zinc total). This was advantageous for the purification process of such a short-lived radionuclide as terbium-149, because large quantities of the material would typically necessitate the use of longer columns and larger volumes, which would have substantially increased the processing time. In addition, zinc is known for being a critical impurity for radionuclides because it greatly affects their radiolabeling capabilities to DOTA chelator^45^. Other implantation options are being considered to improve this aspect of the production and collection process. In particular, aluminum was suggested as a better option considering the reduced sputtering effect on the implantation foil^46^, a better coating performance and its lower impact on radiolabeling capabilities than zinc^45^. However, in view of a patient application, consideration must be given to potential toxicity of aluminum and compliance with human administration limits of toxic metals, which would require additional testing^47–49^.

The radiochemical separation method presented in this work, which allowed an improved quality of terbium-149, was based on Sykam cation exchange resin and LN3 extraction resin, respectively. The Sykam column was applied for separation, while the second column enabled terbium to be concentrated, the α-HIBA to be rinsed out and the product to be eluted in the desired volume of dilute HCl. This procedure has been routinely used in other terbium radionuclide separations published by our group^38,39^. A critical optimization measure involved implementing an initial NH_4_NO_3_ column rinse on the Sykam column to enhance the separation of terbium-149 from zinc. As a result, the [^149^Tb]TbCl_3_ final solution was suitable for the radiolabeling of peptides for preclinical purposes at AMA up to 50 MBq/nmol. This molar activity was more than eightfold greater than that of previous campaigns, where only 6 MBq/nmol was achieved^15^. Terbium-149 high chemical purity was further confirmed by the ICP-MS studies on [^149^Tb]TbCl_3_ samples taken at EOS, which only detected ppb quantities of lead, copper, iron, and zinc. Nevertheless, most of those elements are significant environmental contaminants that are difficult to assess in such low concentrations due to the high risk of external contamination and probable variation in the background, which leads to the variability of the results. Despite that, such amount of contamination did not affect the labeling capacity of the radionuclide, as the [^149^Tb]TbCl_3_ solution at EOS exhibited consistent capacity of radiolabeling independently of the eluted fraction, demonstrating good reproducibility of the separation and low variability during the final elution step. The radiochemical separation was also effective for the separation of the radiolanthanides in question, as shown by the ICP-MS studies (lanthanides < LOQ), and by the γ-ray spectra obtained from the [^149^Tb]TbCl_3_ solutions at EOS (RNP > 99.8%). On the basis of the RNP and AMA achieved with [^149^Tb]Tb-DOTATATE, the quality of terbium-149 was considered adequate for preclinical applications, and was used for in vitro and in vivo studies that will be reported elsewhere. A PET/CT in vivo image was reported in this study to demonstrate the successful preclinical application of [^149^Tb]Tb-DOTATATE.

In addition, this study, along with the previous works published by our group^15–17^, is aimed to form part of a “lighthouse project” for the IMPACT large facility proposal, submitted by PSI and Swiss universities (ETH Zurich and University of Zurich). It foresees the construction of an ISOL facility using a portion of PSI's HIPA facility's high-intensity, high-energy proton beam (100 µA, 590 MeV), comprising an online mass separator connected to dedicated high-power spallation targets for the production of radionuclides (Targeted Alpha Tumour Therapy and Other Oncological Solutions–TATTOOS)^50^. The TATTOOS facility, which will operate with a 50-fold-larger proton beam current than CERN-ISOLDE, is anticipated to produce significantly higher activities, potentially for clinical application. In particular, it is calculated to reach saturation activities ≈1 TBq terbium-149 in the target^51,52^. Nevertheless, this numerical value does not account for factors such as ionization efficiency and release efficiency (including diffusion and effusion delay), which significantly influence the outcome^53^. Compared to CERN-ISOLDE the lower proton beam energy is expected to lead to lower terbium-149 production cross-sections, but the production of disturbing A = 133 pseudo-isobars would be completely suppressed^52^, i.e. the load of the ion source and implantation matrix with cerium isotopes would be avoided. In addition, considering that the radiochemical separation would occur on the same site as the implantation, decay times due to transport would be prevented. Employing the radiochemical separation established in this work, the quality of the final product is also expected to be adequate for future clinical use. This is due to the capacity of obtaining increased activity concentrations, resulting in the possibility to achieve radiolabeling at higher AMA and greater relative chemical purity (with lower impurity levels per GBq), possibly meeting the standard requirements outlined in the European Pharmacopoeia for other radionuclides^40,54^.

## Conclusions

In this study, the production and radiochemical purification of terbium-149 were optimized to provide terbium-149 in quantity and quality, to our knowledge, never achieved in the past, and sufficient for use in more extensive therapeutic preclinical studies than those previously conducted. Particularly, this run of experiments in the PSI/ISOLDE collaboration proved to be the most successful to date and these findings will prove useful for laying the groundwork for future research in radionuclide production using the ISOL technique. This approach will be further developed within the framework of PSI/ETH Zurich/University of Zurich's IMPACT/TATTOOS large-facility project.

### Supplementary Information

Supplementary Information.

## Data Availability

The datasets generated during and/or analyzed during the current study are available from the corresponding author on reasonable request.
