# Supplementary material for: Terbium-149 production: a focus on yield and quality improvement towards preclinical application
Source: Sci Rep. 2024 Feb 8;14:3284. doi: 10.1038/s41598-024-53610-2 (PMC10853284; doi:10.1038/s41598-024-53610-2)
Supplement: Supplementary file 1 — Supplementary Information. [file 41598_2024_53610_MOESM1_ESM.pdf]

Terbium-149 production: a focus on yield and quality improvement towards preclinical application

Supplementary information

C. Favaretto[1,2], P. V. Grundler[2], Z. Talip[2], U. Köster[3,4], K. Johnston[4], S. D. Busslinger[2], P. Sprung[5], C. C. Hillhouse[6], R. Eichler[6,7], R. Schibli[2,8], C. Müller[2,8], N. P. van der Meulen[2,6*]

[1] Nuclear Medicine Department, Universtiätsspital Basel, Basel, Switzerland

[2] Center for Radiopharmaceutical Sciences ETH-PSI, Paul Scherrer Institute, Villigen-PSI, Switzerland

[3] Institute Laue-Langevin, Grenoble, France

[4] Physics Department, ISOLDE/CERN, Geneva, Switzerland

[5] Department Hot Laboratory, Paul Scherrer Institute, Villigen-PSI, Switzerland

[6] Laboratory of Radiochemistry, Paul Scherrer Institute, Villigen-PSI, Switzerland

[7] Department of Chemistry, Biochemistry and Pharmaceutical Sciences, University of Bern, Bern, Switzerland

[8] Department of Chemistry and Applied Biosciences, ETH Zurich, Zurich, Switzerland

†Dedicated to the memory of Gerd-Jürgen Beyer, the “Godfather of terbium-149”

*Corresponding Author: Dr. Nicholas P. van der Meulen

Laboratory of Radiochemistry/Center for Radiopharmaceutical Sciences

Paul Scherrer Institute

5232 Villigen-PSI

Switzerland

Tel: +41 56 310 50 87

email: nick.vandermeulen@psi.ch

Supplementary Notes and Methods

Terbium-149 coproduced isobars and pseudo-isobars

Supplementary Table S1 Decay properties of terbium-149 and its isobars or decay products dysprosium-149, gadolinium-149 (decay product), europium-149 and europium-145 (decay product).[1,2]

	**Tb-149**	**Dy-149**	**Gd-149**	**Eu-149**	**Eu-145**
**Half-life**	4.1 h	4.2 min	9.3 d	93.1 d	5.9 d
**Main decay mode**	EC + α + $β^+$	EC + $β^+$	EC + $β^+$	EC	$β^+$
**Eγ (keV) (Iγ [%])**	352.2 (29.8)<br>165.0 (26.7)<br>388.6 (18.6)<br>652.1 (16.5)<br>853.4 (15.7)<br>817.1 (11.8)<br>861.9 (7.60)<br>464.9 (5.73)	100.7 (15.3)<br>789.4 (11.6)<br>1776.2 (11.7)<br>653.6 (9.1)<br>106.2 (8.30)<br>1805.8 (7.7)<br>253.6 (6.78)<br>775.1 (2.7)	149.7 (48.4)<br>298.6 (27.9)<br>346.7 (23.7)<br>748.6 (8.3)<br>788.9 (7.3)	327.5 (4.75)<br>277.1 (4.18)	893.7 (66)<br>653.5 (15.0)<br>1658.5 (15)<br>1997.0 (7.2)

Only the lines with Iγ > 5% are reported (except for europium-149).

Supplementary Table S2 Decay properties of terbium-149 pseudo-isobars praseodymium-133, cerium-133 and lanthanum-133.[3]

	**Pr-133**	**Ce-133**	**Ce-133m**	**La-133**
**Half-life**	6.5 min	97 min	5.1 h	3.9 h
**Main decay mode**	EC + $β^+$	EC + $β^+$	EC + $β^+$	EC + $β^+$
**Eγ (keV) (Iγ [%])**	134.3 (100)<br>315.6 (88)<br>465.2 (62)<br>330.9 (51)<br>241.9 (42)<br>362.5 (13.6)<br>645.2 (11.8)<br>183.8 (9.6)<br>303.0 (8.0)<br>318.0 (7.4)<br>496.4 (7.4)<br>149.6 (6.4)<br>537.3 (6.1)<br>223.2 (5.4)	97.3 (46)<br>76.9 (15.9)<br>557.7 (11.4)	477.2 (39.3)<br>510.4 (20.8)<br>58.4 (19.3)<br>130.8 (18.0)<br>784.6 (9.7)<br>87.9 (5.2)	278.8 (2.4)<br>302.4 (1.6)<br>290.1 (1.4)

Only the lines with Iγ > 5% are reported (except for lanthanum-133).

Preclinical PET/CT scan

Five-week-old female, athymic CD1 nude mice were obtained from Charles River Laboratories (Sulzfeld, Germany). After an acclimatization period of at least 7 days, mice were subcutaneously

inoculated with AR42J tumor cells, a rat pancreatic tumor cell line, (5 × 10^6 cells in 100 µL phosphate-buffered saline (PBS)) on the right shoulder as previously reported.[4] PET/CT scans were acquired 10-14 days after tumor cell inoculation. [^{149}Tb]Tb-DOTATATE (5 MBq, 0.5 nmol, prepared as described above and diluted in PBS with 0.05% bovine serum albumin) was injected into a lateral tail vein of the mouse. During the scan, the mouse was anesthetized with a mixture of isoflurane and oxygen. The scans were performed using a small-animal bench-top PET/CT scanner (G8, Perkin Elmer, Massachusetts, U.S.A.[5]), as previously reported,[6] with a set energy window ranging from 150 keV to 650 keV. Static whole-body PET scans of 10 min duration were performed at 2 h after injection of the radiopeptide, followed by a CT scan of ~1.5 min. The acquisition reconstruction of the images was performed using the G8 PET/CT scanner software (version 2.0.0.10). All images were prepared using VivoQuant post-processing software (version 3.5, inviCRO Imaging Services and Software, Boston U.S.). A Gaussian post-reconstruction filter (full width at half maximum = 1 mm) was applied to the images and the scale adjusted by cutting 10% off the lower signal intensity.

Supplementary Data

Improved zinc separation achieved by introducing a column rinse step with 1 M NH_4NO_3

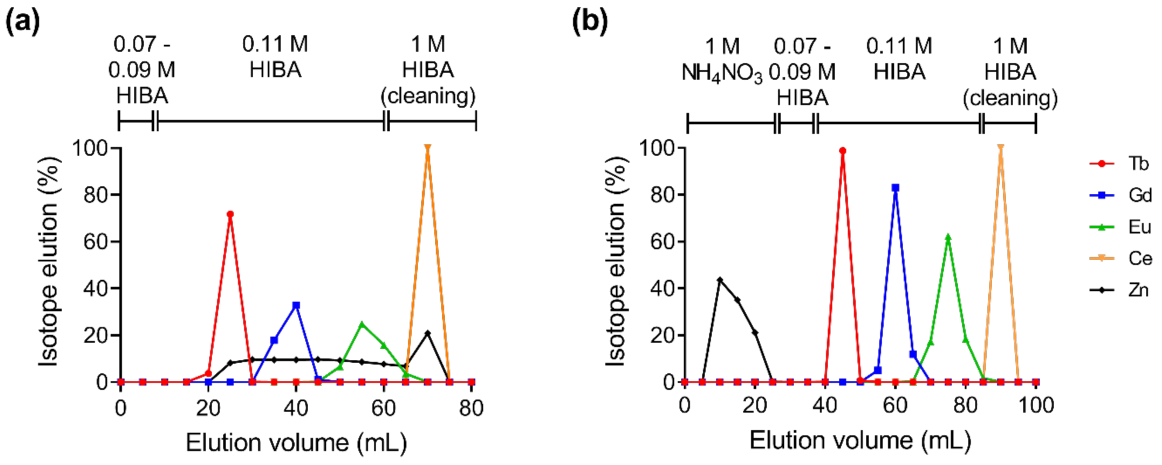

Supplementary Figure S1 (**a**) Representative elution profile of zinc, terbium, gadolinium, europium and cerium from Sykam resin column (1.4 mL) by gradient elution of 0.07–1.0 M α-HIBA. (**b**) Representative elution profile of zinc, terbium, gadolinium, europium and cerium from Sykam resin column (1.4 mL) using 20 mL 1.0 M NH4NO3 followed by gradient elution 0.07–1.0 M α-HIBA.

Residual activity measurement on the gold foil at end of separation

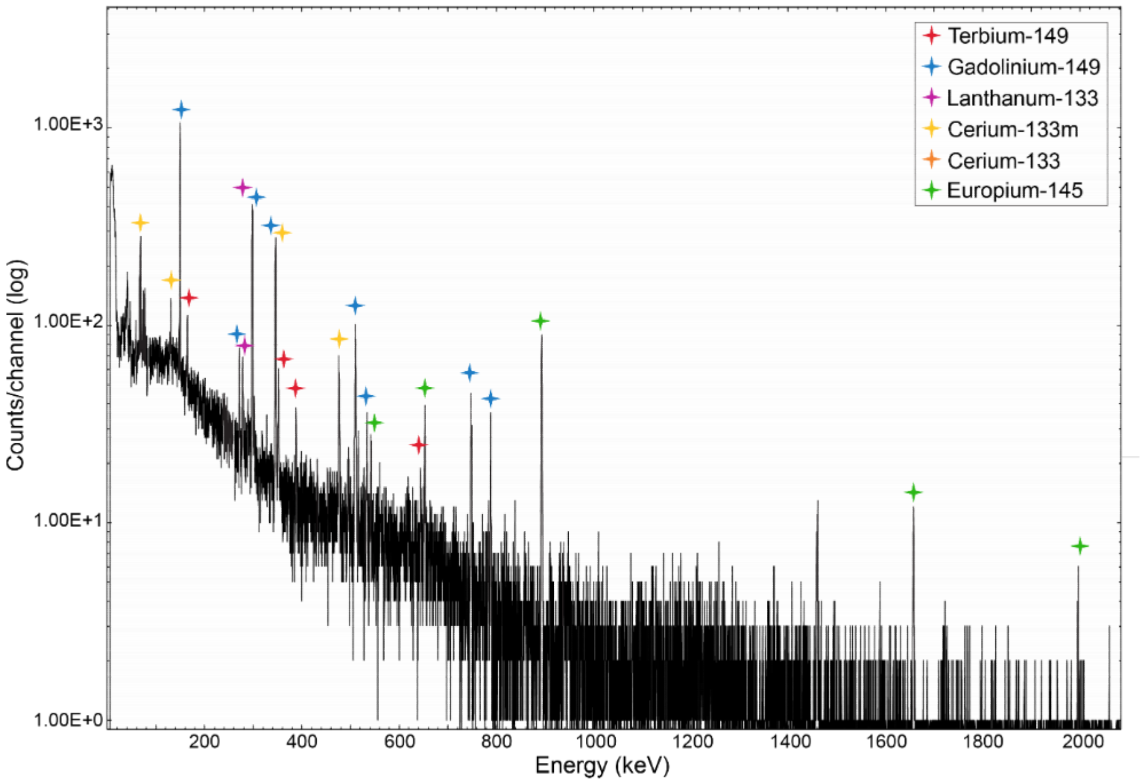

Supplementary Figure S2 γ-spectrum of the gold foil after the dissolution of the Zn layer (90 min measurement time starting 22 hours after EOS; 1 m distance from the detector). Red star = terbium-149 peaks, blue star = gadolinium-149 peaks, violet star = lanthanum-133 peaks, yellow star = cerium-133m peaks, orange star = cerium-133 peaks, green star = europium-145 peaks.

Metals quantification via ICP-MS in [^{149}Tb]TbCl$_3$ samples at EOS

Supplementary Table S3 Zinc, copper, iron and lead contents in 5 samples of [^{149}Tb]TbCl$_3$ at EOS.

Campaign	Production	Zinc (ppb)	Copper (ppb)	Iron (ppb)	Lead (ppb)
November 2021	2 – Fraction 1	120 ± 2.5%	6.40 ± 13.4%	449 ± 1.9%	10.6 ± 20.6%
	2 – Fraction 2	263 ± 2.1%	22.3 ± 6.5%	184 ± 5.7%	814 ± 1.6%
	3 – Fraction 1	73.0 ± 1.9%	2.20 ± 12.7%	31.9 ± 5.7%	22.0 ± 4.1%
	4 – Fraction 1	153 ± 2.4%	< LOQ (5.7)	225 ± 5.6%	422 ± 1.8%
March 2022	4 – Fraction 2	96.4 ± 1.9%	6.90 ± 6.2%	48.5 ± 5.6%	4.87 ± 4.9%

LOD= limit of detection

References

1. Singh, B. & Chen, J. Nuclear Structure and Decay Data for A=149 Isobars. *Nucl. Data Sheets* **185**, 2–559 (2022).

2. Browne, E. & Tuli, J. K. Nuclear Data Sheets for A = 145. *Nucl. Data Sheets* **110**, 507–680 (2009).

3. Khazov, Y., Rodionov, A. & Kondev, F. G. Nuclear Data Sheets for A = 133. *Nucl. Data Sheets* **112**, 855–1113 (2011).

4. Borgna, F. *et al.* Simultaneous visualization of ^{161}Tb-and ^{177}Lu-Labeled somatostatin analogues using dual-isotope SPECT imaging. *Pharmaceutics* **13**, 1–13 (2021).

5. Gu, Z. *et al.* Performance evaluation of G8, a high-sensitivity benchtop preclinical PET/CT tomograph. *J. Nucl. Med.* **60**, 142–149 (2019).

6. Umbricht, C. A. *et al.* Alpha-PET for Prostate Cancer: Preclinical investigation using ^{149}Tb-PSMA-617. *Sci. Rep.* **9**, 1–10 (2019).
